# Supplementary material for: From sequence to enzyme mechanism using multi-label machine learning
Source: BMC Bioinformatics. 2014 May 19;15:150. doi: 10.1186/1471-2105-15-150 (PMC4229970; doi:10.1186/1471-2105-15-150)
Supplement: Additional file 2 — Java code of ml2db. Additional file ml2db_code.tar.gz contains the Java source code to run the multi-label machine learning experiments and save the results to database. The code’s Javadoc is included. [file 1471-2105-15-150-S2.zip › additional file 2/ml2db/ml2db/doc/main/ml2db/mulan/mulanarff/AttributeUtils.html]

AttributeUtils


JavaScript is disabled on your browser.


- Overview
- Package
- Class
- Use
- Tree
- Deprecated
- Index
- Help

- Prev Class
- Next Class

- Frames
- No Frames

- All Classes

- Summary:
- Nested |
- Field |
- Constr |
- Method

- Detail:
- Field |
- Constr |
- Method


main.ml2db.mulan.mulanarff

## Class AttributeUtils

- java.lang.Object
- - main.ml2db.mulan.mulanarff.AttributeUtils

- ---

    

  ```
  public class AttributeUtils
  extends java.lang.Object
  ```

- - ### Constructor Summary

    Constructors

    Constructor and Description

    `AttributeUtils()`
  - ### Method Summary

    Methods

    | Modifier and Type | Method and Description |
    | --- | --- |
    | `static java.util.ArrayList<weka.core.Attribute>` | `fromEnumerationToArrayList(java.util.Enumeration<weka.core.Attribute> enumeration)` |
    | `static java.util.Vector<weka.core.Attribute>` | `fromEnumerationToVector(java.util.Enumeration<weka.core.Attribute> enumeration)` |

    - ### Methods inherited from class java.lang.Object

      `equals, getClass, hashCode, notify, notifyAll, toString, wait, wait, wait`

- - ### Constructor Detail


    - #### AttributeUtils

      ```
      public AttributeUtils()
      ```
  - ### Method Detail


    - #### fromEnumerationToArrayList

      ```
      public static java.util.ArrayList<weka.core.Attribute> fromEnumerationToArrayList(java.util.Enumeration<weka.core.Attribute> enumeration)
      ```


    - #### fromEnumerationToVector

      ```
      public static java.util.Vector<weka.core.Attribute> fromEnumerationToVector(java.util.Enumeration<weka.core.Attribute> enumeration)
      ```


- Overview
- Package
- Class
- Use
- Tree
- Deprecated
- Index
- Help

- Prev Class
- Next Class

- Frames
- No Frames

- All Classes

- Summary:
- Nested |
- Field |
- Constr |
- Method

- Detail:
- Field |
- Constr |
- Method
